# SUMO-Enriched Proteome for *Drosophila* Innate Immune Response

**DOI:** 10.1534/g3.115.020958

**Published:** 2015-08-18

**Authors:** Mithila Handu, Bhagyashree Kaduskar, Ramya Ravindranathan, Amarendranath Soory, Ritika Giri, Vijay Barathi Elango, Harsha Gowda, Girish S. Ratnaparkhi

**Affiliations:** * Indian Institute of Science Education and Research, Pune, India; † Institute of Bioinformatics, 566 066 Bangalore, India

**Keywords:** SUMO, immunity, signaling, NF-kappaB, proteome, regulation

## Abstract

Small ubiquitin-like modifier (SUMO) modification modulates the expression of defense genes in *Drosophila*, activated by the Toll/nuclear factor-κB and immune-deficient/nuclear factor-κB signaling networks. We have, however, limited understanding of the SUMO-modulated regulation of the immune response and lack information on SUMO targets in the immune system. In this study, we measured the changes to the SUMO proteome in S2 cells in response to a lipopolysaccharide challenge and identified 1619 unique proteins in SUMO-enriched lysates. A confident set of 710 proteins represents the immune-induced SUMO proteome and analysis suggests that specific protein domains, cellular pathways, and protein complexes respond to immune stress. A small subset of the confident set was validated by in-bacto SUMOylation and shown to be bona-fide SUMO targets. These include components of immune signaling pathways such as Caspar, Jra, Kay, cdc42, p38b, 14-3-3ε, as well as cellular proteins with diverse functions, many being components of protein complexes, such as prosß4, Rps10b, SmD3, Tango7, and Aats-arg. Caspar, a human FAF1 ortholog that negatively regulates immune-deficient signaling, is SUMOylated at K551 and responds to treatment with lipopolysaccharide in cultured cells. Our study is one of the first to describe SUMO proteome for the *Drosophila* immune response. Our data and analysis provide a global framework for the understanding of SUMO modification in the host response to pathogens.

The innate immune response serves as the first line of defense to combat a wide range of microbial pathogens in multicellular organisms. The process involves the recognition of specific pathogen-associated molecular patterns by cellular receptors that trigger downstream effector responses (Akira *et al.* 2006; Iwasaki and Medzhitov 2010; Hajishengallis and Lambris 2011; Kawai and Akira 2011). *Drosophila melanogaster*, like all invertebrates, lacks an adaptive immune response, defending itself using innate immune-based mechanisms. The *Drosophila* genome codes for immune signaling pathway components similar to those that involved in mammalian innate immunity (Rubin *et al.* 2000; Ferrandon *et al.* 2007; Sackton *et al.* 2007; Hetru and Hoffmann 2009; Ramet and Hultmark 2013). The fruit fly serves as an ideal model system to study innate immunity because of the availability of a plethora of genetic tools. *Drosophila* immune response can be broadly classified into three categories, namely a cellular response, melanization with wound repair, and the humoral response, which causes the production of a battery of antimicrobial peptides (AMPs) by the fat body and hemocytes. Infection leads to the activation of the Toll (TL) pathway and immune-deficient (IMD) signaling cascades, which in turn leads to the translocation of nuclear factor (NF)-κB factors dorsal (DL), dorsal-like immune factor (Dif), and relish (REL) into the nucleus, leading to transcriptional activation of defense genes (Anderson 2000; De Gregorio *et al.* 2001; Agaisse and Perrimon 2004; Hetru and Hoffmann 2009). The Jun kinase (JNK), Janus kinase/signal transducers and activators of transcription (JAK-STAT), and Ras/mitogen-activated protein kinase (MAPK) pathways also play important roles either in modulating the immune responses (Boutros *et al.* 2002; Agaisse and Perrimon 2004; Delaney *et al.* 2006; Chen *et al.* 2010; Ragab *et al.* 2011) or work independently to activate certain effector responses like apoptosis, stress, and increased hemocyte proliferation.

Reversible post-translational modifications (PTMs) of proteins through addition and removal of molecular moieties are essential in exerting rapid changes to external stimuli without the input of transcription, protein synthesis, and subsequent turnover of messenger RNA and protein. Recently, covalent modification of proteins by the small ubiquitin-like modifier (SUMO) has emerged as an important PTM mechanism in regulating transcription, translation, cell cycle, DNA replication and repair, and other basic cellular process (Muller *et al.* 2001; Hay 2005; Geiss-Friedlander and Melchior 2007). In *Drosophila*, there is a single SUMO gene, *Smt3* (Bhaskar *et al.* 2000). The addition of SUMO to its target proteins occurs with the assistance of the E1, E2, and E3 enzymes similar to that of the ubiquitin pathway, the enzymatic machinery however being distinct from the ubiquitin cycle. The attachment of SUMO to target proteins usually occurs at a consensus site ψKXE (where ψ is a hydrophobic residue, most often I, L or V) (Desterro *et al.* 1999; Muller *et al.* 2001; Rodriguez *et al.* 2001; Sampson *et al.* 2001; Hendriks *et al.* 2014). SUMOylation, is however, also found routinely to occur at nonconsensus sequences, indicating that the SUMOylation signature motif may be more complex and dependent on other factors such as other post-translational modifiers (Hietakangas *et al.* 2006), other linear motifs (Hendriks *et al.* 2014), or a three-dimensional structural motif. Also, demonstrating SUMOylation in strong consensus (ψKXE) sites is sometimes challenging, indicating that the presence of a consensus motif is probably not a sufficient determinant for SUMOylation. The presence of SUMO interaction motifs (SIMs) on other proteins provides surfaces for direct noncovalent interaction with SUMOylated proteins or may aid SUMO modification of the protein (Desterro *et al.* 1999; Sampson *et al.* 2001; Kerscher 2007; Zhu *et al.* 2008).

A number of proteins in the mammalian and fly immune signaling cascades have been shown to be regulated by PTM mechanisms such as phosphorylation and ubiquitination (Rutschmann *et al.* 2000; Silverman *et al.* 2000; Zhou *et al.* 2005b; Silva *et al.* 2007). SUMOylation has been predicted to be involved extensively in the regulation of immune processes (Mabb and Miyamoto 2007), but very few proteins have been shown to be physically SUMOylated (Desterro *et al.* 1998; Bhaskar *et al.* 2002; Huang *et al.* 2003; Fukuyama *et al.* 2013). Previous studies in *Drosophila* show the role of SUMO and its conjugation pathway component *Ubc9* in AMP response, phagocytosis, and hemocyte proliferation (Chiu *et al.* 2005; Paddibhatla *et al.* 2010). In *Drosophila* S2 cells and larvae, knockdown of SUMO conjugating enzyme Ubc9 affects the production of AMPs (Bhaskar *et al.* 2002; Chiu *et al.* 2005); the Ubc9 mutant larvae also show overproliferation of hemocytes as well as the presence of melanotic tumors in the hemolymph (Chiu *et al.* 2005). *Ubc9*-heterozygous flies are more susceptible to infection with *Escherichia coli* compared with wild type (Fukuyama *et al.* 2013). In *Drosophila*, DL is the only protein demonstrated to be SUMOylated in TL/NF-κB pathway (Bhaskar *et al.* 2002), although there is a clear evidence for SUMO regulation of TL signaling (Bhaskar *et al.* 2000; Chiu *et al.* 2005; Smith *et al.* 2011; Anjum *et al.* 2013), whereas IRD5 has been shown to be SUMOylated in the IMD pathway (Fukuyama *et al.* 2013).

In this study, we characterize the role of SUMO modification in regulating the *Drosophila* innate immune response in cultured Schneider (S2) cells. *Drosophila* S2 cells efficiently demonstrate both the cellular and humoral arms of the innate immune response by phagocytosing microorganisms and also secreting AMPs in response to treatment with lipopolysaccharide (LPS), mimicking an immune challenge (Bhaskar *et al.* 2000, 2002; Smith *et al.* 2004). S2 cells have been used routinely to understand the immune response in a number of studies (Ramet *et al.* 2002; Gronholm *et al.* 2012; Zhu *et al.* 2013; Tsuzuki *et al.* 2014) and are especially useful for genome-wide RNA interference (RNAi) screens (Kim *et al.* 2010; Kuttenkeuler *et al.* 2010; Qin *et al.* 2011). First, we show that in S2 cells, in the absence of SUMOylation, transcriptional profiles of defense genes activated by NF-κBs are modulated. Second, we identify immune-responsive quantitative proteomics changes in the cell with respect to SUMOylation. A confident list of 710 proteins that comprise the LPS-induced SUMO-enriched proteome is identified that includes previously described SUMO substrates as well as novel SUMO targets and implicates specific protein complexes in the immune response. Bioinformatic analysis of this list indicates enrichment of a diverse set of cellular processes that in turn suggests that the immune response is complex and multidimensional. Third, we validate a dozen proteins as *bona fide* SUMO targets by demonstrating physical SUMOylation. One key target studied, Caspar (Casp), the *Drosophila* analog of human FAF1, and a negative regulator of IMD/NF-κB signaling, is further validated as a SUMO target in cultured cells.

## Materials and Methods

### S2 cell culture, LPS treatment, and RNAi

#### 529SU cells:

The stably transfected 529SU cell line expressing full length FLAG-*smt3(SUMO)*, HA-*ubc9* under the control of a metallothionin promoter was a kind gift from the Courey Lab, UCLA (Bhaskar *et al.* 2002). This line was used for RNAi and affinity pulldown experiments, whereas both 529SU cells and S2 cells were used for validation and control experiments. Expression of the SUMO cycle components in the cell line was validated by Western blots using Rb anti-SUMO (1:5000, Courey Lab), Rb anti-FLAG (Sigma, 1:1000), and Rb anti-HA (Millipore, 1:000). The stocks of the cells were maintained in the presence of (300 μg/mL) hygromycin, in the absence of antibiotics, in *Drosophila* Schneider cell medium (Sigma-Aldrich) complemented with heat-inactivated 10% fetal bovine serum (Gibco) at 24°. For large-scale experiments, hygromycin was not added to the medium.

#### Induction of the immune response:

Crude LPS (0111:B4, batch 129K4025; Sigma-Aldrich) was added to the culture medium at a final concentration of 10 μg/mL to induce a comprehensive immune response. Unlike purified LPS, crude LPS activates both the TL/NF-κB and IMD/NF-κB signaling networks (Kaneko *et al.* 2004). Because these cells were used for downstream proteomic analysis, the LPS-mediated induction of both the TL and IMD pathway components was characterized by harvesting cells at different time points, ranging from 0 to 24 hr (Supporting Information, Figure S1B). Quantitative real-time polymerase chain reaction (qRT–PCR) analysis of the transcripts of AMP genes *Drosomycin (Drs)*, *Diptericin (Dipt)*, *Defensin (def)*, *Attacin-AB (AttAB)*, *Attacin-D (AttD)*, *Cecropin A* (*CecA*), *Metchnikowin* (*Mtch*), *Drosocin* (Dros), *Tl*, *Spazle (Spz)*, *Transferrin-3(Tsf3)*, *REL*, *smt3 (SUMO)*, *lesswright (ubc9)*, *Sumo-activating enzyme 1 (SAE1/Aos1)* and *Sumo –activating enzyme 2 (SAE2/Uba2)*, along with control transcript *ribosomal protein 49* (*rp49*) and *ribosomal protein 32* (*rp32*), were collected. 529SU cells do not need hormonal supplements for activation and give a robust and reproducible immune response (Bhaskar *et al.* 2002) in response to crude LPS. On the basis of the time-dependent expression profile of the AMP genes, we chose the 3-hr time point as a single time point for quantitative proteomic analysis.

#### RNAi, RNA isolation, and qRT-PCR:

Double-stranded RNA (dsRNA) was generated by *in vitro* transcription (Megascript kit; Ambion). For RNA interference assay, 1 mL of high-density cells were split (1:5 v/v) and plated in 12-well plates at a count ∼1 × 10^6^ and allowed to settle for 20 min. Next, the old medium was completely removed and substituted with serum-free media to which 10 µg of relevant dsRNA/mL was added and the cells were incubated for 2 hr. The serum-free media was then replaced by serum-containing media and allowed to grow. After 72 hr, one set of cells was treated with 10 µg/mL LPS for 2−24 hr while sterile water was added to control cells. Post infection, the cells were centrifuged, washed, and total RNA was isolated using TRIzol (Invitrogen). After degrading any genomic DNA with RNase-free DNase (Promega), we re-precipitated the RNA, and 1 μg was subjected to reverse transcription-PCR with poly dT primers (for SYBR experiments) or by random primers (TaqMan Assay) to obtain complementary DNA (cDNA). This cDNA was used to check for the levels of AMPs and SUMO pathway components using rp49 as a relative housekeeping control. For western blot analysis after RNAi, these cells were induced with 0.5 mM CuSO_4_ to express FLAG-SUMO-GG 48 hr before treatment with LPS. The cells were further collected and lysed in Nu-SDS PAGE buffer followed by sodium dodecyl sulfate-polyacrylamide gel electrophoresis (SDS-PAGE) and Western blot analysis using Rb anti-FLAG antibody (1:1000; Sigma-Aldrich).

#### RT-PCR measurement of kinetic curves for defense genes with and without SUMO knockdown:

In 529SU cells we used dsRNA to knock-down SUMO gene transcripts to less than ∼10%, judged by using qPCR as well as using Western Blots (Figure S1A). We monitored transcripts levels in *SUMO*-depleted S2 cells in response to crude LPS over a time period of 0−24 hr (Figure S1B). Transcript levels were monitored with RT-PCR using both SYBR green and TaqMan probes. In response to *SUMO* reduction, *drosomycin (Drs)* failed to activate (Bhaskar *et al.* 2002; Figure S1B) whereas *Transferrin3 (Tsf3)*, *Attacin D (AttD)*, and *REL* levels were lower compared with controls. Reduction of *SUMO* transcripts did not appear to affect the expression of genes coding for *Tl* (Figure S1B) as well as for *Ulp1*, *Sae1*, *Sae2*, or *ubc9* (data not shown). The reasons for the differential regulation of the AMP genes is unclear at this point and may include the possibility of different mechanisms for activation or feedback regulation of different NF-κB targets. Primers used for SYBR assays were Rp49-F, GACGCTTCAAGGGACAGTATC; Rp49-R, AAACGCGGTTCTGCATGAG; Attacin AB-F, GGCCCATGCCAATTTATTCA; Attacin AB-R, CATTGCGCTGGAACTCGAA; CecropinA-F, TCTTCGTTTTCGTCGCTCTC; CecropinA-R, CTTGTTGAGCGATTCCCAGT; Defensin-F, AGGTTCCTTAACCTCCAATC; Defensin-R, CATGACCAGCATTGTTGTAG; Diptericin-F, AGGTGTGGACCAGCGACAA; Diptericin-R, TGCTGTCCATATCCTCCATTCA; Metchnikowin-F,GCTACATCAGTGCTGGCAGA; Metchnikowin-R, AATAAATTGGACCCGGTCT; Drosomycin-F,CGTGAGAACCTTTTCCAATATGATG; Drosomycin-R,TCCCAGGACCACCAGCAT; Drosocin-F, GCACAATGAAGTTCACCATCGT; Drosocin-R, CCACACCCATGGCAAAAAC; Smt3-utr-F, AACCACAAAAGCAAAAACACAAC; and Smt3-utr-R,GTTATTTACGCACACAGACGC whereas probes for TaqMaq were as follows: spz-Dm02151534_g1,Tl-Dm02151201_g1, Dif-Dm01810799_m1, dl-Dm01810803_g1, Rel-Dm02134843_g1, imd-Dm01845288_g1, Drs-Dm01822006_s1, AttA-Dm02362218_s1, AttC-Dm01821390_g1, smt3-Dm02361838_s1, Aos1-Dm02139633_g1, AttD-Dm02135981_g1, Tsf3-Dm01821472_g1, Act88F-Dm02362815_s1, and RpL32-Dm02151827_g1.

### FLAG affinity purification of SUMO conjugates

**Figure 1:**
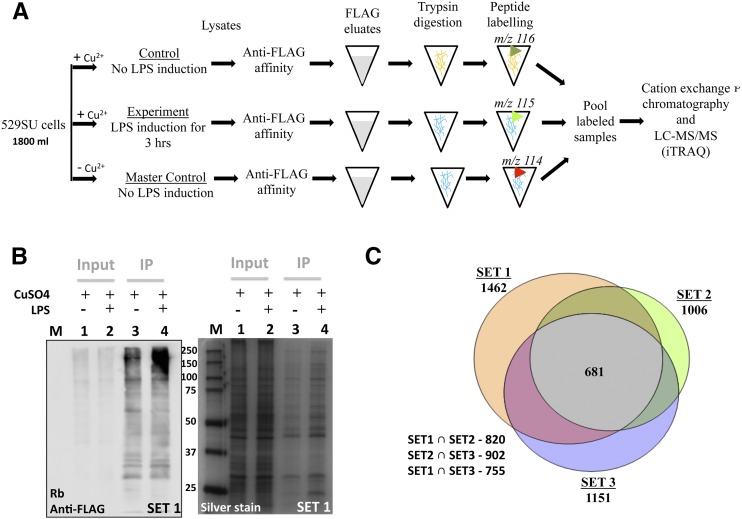
Measuring the small ubiquitin-like modifier (SUMO)-enriched proteome before and after LPS treatment using quantitative proteomics. (A) Schematic representation of the protocol followed for isobaric tag for relative and absolute quantitation (ITRAQ) analysis: 529SU cells were treated with or without CuSO_4_ and/or lipopolysaccharide (LPS), lysed, and the supernatant incubated with FLAG beads. A total of 100 μg of the proteins eluted from the FLAG beads were subject to trypsin digestion and labeled by ITRAQ reagents of varying mass. Samples were pooled, separated into fractions by cation exchange chromatography and each fraction was analyzed by LC-MS/MS, as described in *Materials and Methods*. This protocol was used to collect data for three biological replicates. (B) SUMO induction and affinity purification of SUMOylated proteins: 529SU cell lysates, post-LPS treatment were affinity purified using mouse anti-FLAG agarose. Western blot of the Input lysate (Lane 1 and 2) and affinity-purified (Lane 3 and 4) extracts indicate successful induction and affinity purification of SUMOylated proteins. These SDS-PAGE gel are a representation of one (Set 1) of the biological replicates and are loaded with 1% of the input and 5% of the total, affinity purified FLAG elute. M indicates a ladder of molecular weight markers. (C) Unique proteins identified in the biologic replicates: a Venn representation of unique proteins identified in three biologic replicates, with each replicate containing an LPS(−) and LPS(+) sample. Of the 1821 unique proteins found in the experiments, 1619 were processed for bioinformatic analysis after subtraction of the proteins identified in the master control.

The starting point for the proteomics experiments was 1800 mL of 529SU cells at a cell density of ∼1 × 10^6^ cells/ mL, in thirty 300-cm^2^ culture flasks. The cells were split into three 600-mL aliquots (10 flasks each), with two flasks induced with 0.5 mM CuSO_4_ and the third serving as master control (Figure 1A). At 3 days after the split (and CuSO_4_ induction; cell density ∼1 × 10^7^), half of the induced flasks (10 flasks) were mock treated with sterile water, whereas another 10 (induced) flasks were treated with 10 μg/mL LPS for a period of 3 hr. The cells were collected from the flasks near the end of the incubation period, centrifuged at 1000*g*, washed with 1× phosphate-buffered saline, lyzed with RIPA buffer (50 mM Tris, pH 7.4; 1% NP-40; 0.5% Na-deoxycholate; 0.1% SDS; 150 mM NaCl; 1 mM ethylenediaminetetraacetic acid; and 0.01% sodium azide), freshly supplemented with 40 mM *N*-ethylmaleimide (Sigma-Aldrich) and Complete Protease Inhibitor Cocktail (1 tablet per 100 ml; Roche). The entire 1800-mL set was repeated thrice (three biological replicates) on three different weeks.

The suspension was further lysed in a bioruptor (Diagenode, 130W, 15 min total time, 30-sec on/off pulse) and the lysate was centrifuged at 25,000 g at 4° for 45 min. The supernatant was precleared using protein G sepharose (GE Healthcare) for 1 hr at 4°. Equal concentrations of the precleared lysates from uninduced master control, induced without LPS and induced with LPS cells, were nutated with RIPA-equilibrated anti-FLAG agarose (1 mL) at 4° overnight. The next day, the beads were separated from the lysate by centrifugation (1000*g*), washed three times with TBS-T (50 mM Tris, pH 7.4; 150 mM NaCl; and 1% Triton X-100), followed by two washes in TBS. The bound proteins were eluted from the beads using 10 bead volumes of FLAG peptide (Sigma-Aldrich) at a concentration of 200 μg/mL for 4 hr. The eluted proteins were concentrated using amicon concentrator (Millipore) with a pore cut off of 10 kDa. A portion of the eluted proteins was separated by SDS-PAGE followed by Western blot analysis using Rb anti-FLAG antibody (Sigma-Aldrich) and silver staining. The remaining protein elute was dialyzed extensively against 100 mM NH_4_HCO_3_ to remove any TBS and lyophilized multiple times to remove salt for further mass spectrometry (MS) analysis.

### SDS-PAGE gels, Western blots, antibodies, and silver staining

The amount of protein in the samples from affinity pulldowns was quantitated using Lowry reagent (Bio-Rad). A total of 1% of the input and 5% immunoprecipitation (IP) eluate from each set was loaded onto 10% SDS-PAGE gel, which was then transferred to a polyvinylidene fluoride membrane in semi-dry transfer buffer (250 mM Tris, 1.5 M glycine with 5% methanol) at constant 350 mA for 1 hr, 30 min. The membrane was incubated in TBS with 0.1% Tween 20 (TBS-T) and 5% nonfat dry milk for 1 hr. Primary antibodies were diluted in TBS-T/5% milk at 1:1000 for FLAG (Rb, Sigma-Aldrich) and 1:1000 for HA (Mouse, Millipore). The blot was developed using Millipore ECL on LAS 4000 Imager (Fujifilm). For silver staining, the SDS-PAGE gels were fixed in 5:4:1 of ethanol/water/acetic acid. After washing the fixed gels with distilled water, they were sensitized in 0.02% sodium thiosulfate. The gel was further washed briefly with distilled water and kept in 0.2% (w/v) silver nitrate solution for 30 min. The gel was washed thoroughly with Milli-Q and developed with the use of sodium carbonate with sodium thiosulfate and formaldehyde until bands were seen clearly, after which the reaction was stopped using 6% acetic acid.

### Quantitative protein profiling by isobaric tag for relative and absolute quantitation (ITRAQ)

#### ITRAQ labeling and strong cation exchange chromatography fractionation:

Three biological replicates of each set (uninduced master control, induced untreated, induced LPS treated) were processed in the Mass Spectrometry Facility at the Institute of Bioinformatics, Bangalore. For the induced, LPS-treated and induced, LPS-untreated sets, the lyophilized samples were resuspended in water and 100 μg of each sample treated with 2 μL of reducing agent [tris (2-carboxyethyl) phosphine] at 60° for 1 hr and alkylated with cysteine-blocking reagent, methyl methanethiosulfonate for 10 min at room temperature. For the uninduced master control, very little protein was eluted (<5 μg; Figure S1C, Lane 1) and all the material collected was processed for analysis. The samples were digested overnight with sequencing-grade trypsin (Promega, Madison, WI) (1:20) at 37°. Peptides from master control; induced, LPS-untreated; and induced, LPS-treated experimental sets were labeled with ITRAQ reagents that would yield reporter ions of m/z 114, 115, and 116, respectively. Labeled peptides from all three conditions were pooled and fractionated by strong cation exchange chromatography on Poly SULFOETHYL A column (100 × 2.1 mm, 5-µm particles with 300 A pores, PolyLC; Columbia, MD) using a linear gradient of 5–40% Solvent B (350 mM KCl in 10 mM KH_2_PO_4_, 20% acetonitrile, pH 2.8). Fractionated samples were collected, desalted using stage tips vacuum dried, and stored at −80° until liquid chromatography–tandem mass spectrometry (LC-MS/MS) analysis.

#### LC-MS/MS analysis:

LC-MS/MS analysis of ITRAQ-labeled peptides was carried out on an LTQ-OrbitrapVelos mass spectrometer (Thermo Electron, Bremen, Germany) interfaced with Agilent’s 1100 series nanoflow liquid chromatography system (Agilent Technologies, Santa Clara, CA). Peptides from each fraction were enriched and washed on a trap column (75 µm × 2 cm, 5 µm, 120Å, Magic C_18_ AQ; Michrom Bioresource), at a flow rate of 3 µL/min and then resolved on an analytical column (75 µm × 10 cm, 5 µm, 120Å, Magic C_18_ AQ; Michrom Bioresource) at a flow rate of 300 nL/min using a linear gradient of 5–40% solvent B (90% acetonitrile in 0.1% formic acid) over a period of 65 min. The total run time per sample was 85 min. The resolved peptides from analytical column were delivered to mass spectrometer through an emitter tip (8 µm, New Objective, Woburn, MA). LC-MS/MS data were acquired in a data-dependent manner in FT mode. MS spectra were acquired with a window of *m*/*z* 350 to 1800. The 20 most-abundant precursor ions were selected for fragmentation from each MS scan. Data were acquired at MS resolution of 60,000 (m/z 400) and MS/MS resolution of 15,000. Precursor ion fragmentation was carried out using higher-energy collision mode with normalized collision energy of 41%. Monoisotopic precursor selection was enabled and the precursor ions that were selected for fragmentation was dynamically excluded for 50 sec.

#### MS data analysis:

The MS data were analyzed with the Proteome Discoverer software (Thermo Scientific, version 1.3.0.339). The data were searched against Flybase (FB2010_04 Dmel Release 5.27) database containing 43,900 protein sequences along with known contaminants using SEQUEST search algorithm. The parameters used for data analysis included trypsin as a protease (allowed one missed cleavage), ITRAQ labeling at N-terminus and lysine residues, and cysteine modification by methyl methane thiosulfonate as fixed modifications and oxidation of methionine as a variable modification. The precursor and product ion mass error tolerance were fixed at 20 ppm and 0.1 Da, respectively. The precursor range was set at 500−8000 Da. The peptide and protein data were extracted using high peptide confidence (1% false discovery rate) and top one peptide rank filters. The relative abundance of proteins across conditions was determined with the Proteome Discoverer Software (Thermo Scientific) based on difference in the peak intensity of reporter ions in the MS/MS spectra of each peptide that was ultimately used for quantitating corresponding protein. Differentially regulated proteins were represented by the ration of LPS treated/ untreated ITRAQ ratio. Table S1 summarizes the proteins discovered along with the ITRAQ ratios determined in the three sets. Table S2 contains the raw data, which lists the peptides/proteins identified in all three biological replicates (Set 1, Set 2, Set 3).

#### Analysis of the list generated by MS:

The ITRAQ experiment generated a list of 1821 potentially SUMOylated substrates. The final list, after subtraction of the 195 overlapping proteins detected in the uninduced master control, consisted of 1619 unique proteins, many identified in at least two biological replicates (Table S1). This list contains SUMO-enriched proteins including both proteins that are SUMOylated or that interact with SUMOylated proteins. We analyzed the listed proteins using multiple methods as listed below.

#### Biological replicates and ITRAQ ratios:

Three biological replicates were used for the studies, with 923 proteins identified in two or all three replicates. For each peptide/protein, the change in SUMOylation status after LPS induction was quantitated by dividing the LPS induced score for each protein by the uninduced value. The ratio thus indicated the fold increase or decrease in SUMOylation in response to LPS. The variability of the scores across the sets for the same protein was an indicator for biologic variability as well as the noise in the experiment. In general, ITRAQ values of >2 were taken to indicate significant changes in SUMOylation states, leading to a confident set of 710 proteins. For the global analyses, the confident set was used. The logic of using a twofold threshold is as follows; a confident dataset of 154 SUMOylated proteins in *Drosophila* early development has been published (Nie *et al.* 2009). Also available in literature are individual studies that demonstrate certain proteins to be SUMOylated. Taken together, we collected a list of 118 proteins that also were common to our dataset. ITRAQ ratios of these proteins, as measured in our experiment, were tabulated and it was found that values ranged from 1.63 to 4.15, with 85% of proteins (100/118) having an ITRAQ ratio of >2-fold. Thus, -fold values >2 represented a reasonable biological threshold to identify a confident set of SUMO-enriched proteins.

#### Analysis of the confident set and Gene Ontology:

To investigate the affect of LPS treatment on different biologic processes in 529SU cells, enrichment analysis was performed by comparing data using the PANTHER Classification system 10.0 (Thomas *et al.* 2003) and tools from the DAVID Bioinformatics resources 6.7 (Huang da *et al.* 2009a,b). GO analysis was performed on *Drosophila melanogaster* gene database as the reference list. For PANTHER the *P*-value listed is calculated by the program using the binomial test for each category with a Bonferroni correction. For DAVID the *P*-value calculation is based on a modified Fisher exact test.

#### Cytoscape analysis and SUMO, SUMO binding motif prediction:

The SUMO proteomic list was compared to the *Drosophila* Proteome interaction Map, DPiM (Guruharsha *et al.* 2011) using a DPiM input file generated by the authors (File S1; Guruharsha *et al.* 2011). The *Drosophila* interactome was displayed using the open source bioinformatics software platform Cytoscape (Cline *et al.* 2007). The SUMOylation site prediction the SUMO binding site (SIM) prediction was carried out using JASSA (http://www.jassa.fr/) (Beauclair *et al.* 2015), with the threshold criteria set at “only consensus” for SUMO sites and “high-cut-off” for SIM prediction. Protein complexes described in S2 cells (Guruharsha *et al.* 2011), and also as categorized in Flybase (Flybase Consortium 2003), were listed and analyzed for SUMOylated elements.

### Validation of SUMO conjugation

The ITRAQ experiment generated a list of potential SUMOylated. A subset of the 1619 proteins (∼50) could be related to known components of the immune response. Physical SUMOylation of a few targets could be demonstrated by expressing the target protein concomitantly with the SUMO cycle components E1 (SAE1, SAE2) and E2 (Ubc9) and SUMO *in-bacto* (Nie *et al.* 2009). Target proteins were obtained from one or all of the following sources. The entire *Drosophila* Gold cDNA collection was procured from the *Drosophila* Genome Resource Centre (DGRC), Bloomington, Indiana. Where available, cloned genes were used from this collection; otherwise, the genes were cloned from versions available in DGRC, such as Expressed Sequence Tags or the *Drosophila* Gene Collection ver 1.0, 2.0, or 3.0. All genes used for our studies were sequenced to confirm the gene/ DNA sequence before use.

#### Bacterial expression:

Genes were subcloned into the pGEX-4T1 vector (Promega) for expression in bacteria. To demonstrate physical conjugation of the target protein, proteins were expressed as GST fusions along with components of the *Drosophila* SUMO pathway using the *in-bacto* system (Nie *et al.* 2009). In summary, the GST-substrate fusion was expressed in bacteria along with either 6XHis-FLAG-SUMO-GG, a mature, active form of SUMO, or 6XHis-FLAG-SUMO-ΔGG, which could not conjugate to substrates. Also coexpressed were the *Drosophila* SUMO activation enzymes (SAE1, SAE2) and the SUMO conjugase Ubc9 (E2). If the substrate could be SUMOylated, in a western, post-GST affinity pull down, one would see expression of the substrate protein (using Rb Anti-GST antibody, 1:5000; Santa Cruz Biotechnology) in both the SUMO-GG and SUMO-ΔGG lanes, but only the SUMO-GG lane would show additional bands >15 kDa above the main band. These additional bands would cross react with a anti-6X-His antibody (Mouse Anti-His, 1:3000) or a Rabbit anti-SUMO antibody (1:5000). The isopropyl β-D-1-thiogalactopyranoside induction method was used at a temperature range of 18−37° to locate conditions of soluble expression for SUMO cycle components as well as the GST-fusion protein.

#### Expression in 529SU or S2 cells:

Casp was procured from DGRC (FMO05904), which had C-terminal FLAG and 6X-His tags and also subcloned into pRM-HA3 both untagged and also with a N-terminal 6XHis tag. All constructs were sequenced after subcloning and before use. QIAGEN Midipreps were used to generate plasmid DNA (1 μg/μL) that was transfected into cells using Mirus *TransIT* transfection agent based on the manufacturer’s recommendations. Cell lysis, FLAG or HA affinity, SDS-PAGE gels, IPs, and Western blots were carried out or processed as per protocols described earlier in this section. For Casp IP experiments, cells were transfected in a single flask at 50% confluency, and 0.5 mM CuSO_4_ was added on the same day. After 48 hr, allowing for Casp expression, equal number of cells were split into a 12-well plate, 0.5 mL cells per well, allowing for multiple experiments at the same transfection efficiency. Cells (529SU or S2) were then heat shocked (1 hr, 37°, or treated with LPS (10 μg/mL; 1−6 hr). For the Casp/LPS induction experiments with 529SU cells, equal amount of protein, as measured by the Bradford assay was loaded into each well.

### Data availability

Figure S1 contains supporting figures. Table S1 contains processed ITRAQ data with subsections that lists stepwise, process followed to delineate the confident set of 710 proteins. Table S2 is a zipped file contains three xls files (Set1, Set2, and Set3). The files contain tables with data on peptides identified; Gene Symbol, Description Score, Coverage, and ITRAQ fold change.

## Results

### Quantitative proteomics of the SUMO-enriched proteome

To measure the quantitative changes in SUMOylation levels, in response to an immune challenge, we used *Drosophila* S2 cells in conjunction with ITRAQ proteomics. 529SU, a stable S2 cell line that expresses FLAG-SUMO and HA-Ubc9 (Bhaskar *et al.* 2000, 2002; Smith *et al.* 2004), was used as tagged SUMOylated proteins could be efficiently purified by affinity chromatography from cellular lysates.

Before the start of the proteomics experiments, the 529SU cell line was thoroughly validated. 529SU cells expressed FLAG-SUMO in response to CuSO4 induction. *SUMO* knockdowns led to global decrease in SUMOylation (Figure S1A), whereas *Ulp1* knockdown increased global SUMOylation. Kinetic data for changes in levels of AMPs transcripts in response to treatment with LPS (see *Materials and Methods*) also was collected before and after SUMO knockdown for about a dozen transcripts (see *Materials and Methods*). Crude LPS contains contaminants that lead to a broad activation (Kaneko *et al.* 2004) of immune response including targets of both TL/NF-κB and IMD/NF-κB signaling networks for both 529SU and S2 cells. The data collected agrees with published results in S2 cells (Bhaskar *et al.* 2002) and flies (Chiu *et al.* 2005; Fukuyama *et al.* 2013), which suggest that a subset of defense genes show reduced or delayed activation in the absence of SUMO (Figure S1B).

The experimental protocol for the proteomics studies was as in Figure 1A. Experiments were carried out in three biologic replicate sets (Set 1, 2, and 3). Each set contained three samples (600 mL of cells each) of 529SU cells, with uninduced “master control,” 0.5 mM CuSO_4_-induced “control” without LPS, and induced ”experiment” with 10 μg/mL LPS. A fraction (5%) of the lysates (“input”) and elute from mouse anti-FLAG-agarose (IP) is loaded on a SDS-PAGE gels, for Western and Silver Stain analysis (Figure 1B). The master control, without CuSO_4_ induction, on IP shows very little protein (Figure S1C, Lane 1) and does not react with the Rb-FLAG antibody. The lanes with FLAG-SUMO induction have characteristic SUMOylated species laddered on the gel. The cells treated with LPS show an increase in global SUMOylation (Figure 1B, Lane 4) more obvious for proteins >100 kDa. The three biologic replicates (Set 1, 2, and 3) were processed for ITRAQ proteomics as described in *Materials and Methods*.

### Analysis of the SUMO-enriched proteome

An analysis of the SUMO enriched proteome over the three replicates indicates that in total 1821 unique proteins were identified using a cutoff of 95% probability for identification of peptides (Figure 1C; Table S2). Of these 681 (37%) were common to all three sets and 1111 (or 61%) common to at least 2 sets. The LPS-treated/control ITRAQ ratios vary from 0.3 to 6 for the data points collected (Table S2). As is well documented, the ratios measured by the ITRAQ experiment are compressed (Karp *et al.* 2010) and a relative measure rather than the actual fold values of changes in SUMOylation. After subtraction of the common proteins identified in the master control Set (Table S1), 1619 proteins are taken forward for analysis. Of these, 100 proteins (66%) are common with the published SUMO proteome (a list of 150 proteins) from 0- to 3-hr *Drosophila* embryos (Nie *et al.* 2009) and approximately 18–25% of the proteins are represented in SUMO proteomes from other organisms (Vertegaal *et al.* 2004; Wohlschlegel *et al.* 2004; Denison *et al.* 2005; Golebiowski *et al.* 2009; Kaminsky *et al.* 2009; Hendriks *et al.* 2014). This is in the range seen for overlapping independent SUMOylation data sets as analyzed earlier (Hendriks *et al.* 2014) for mammalian cells.

**Figure 2:**
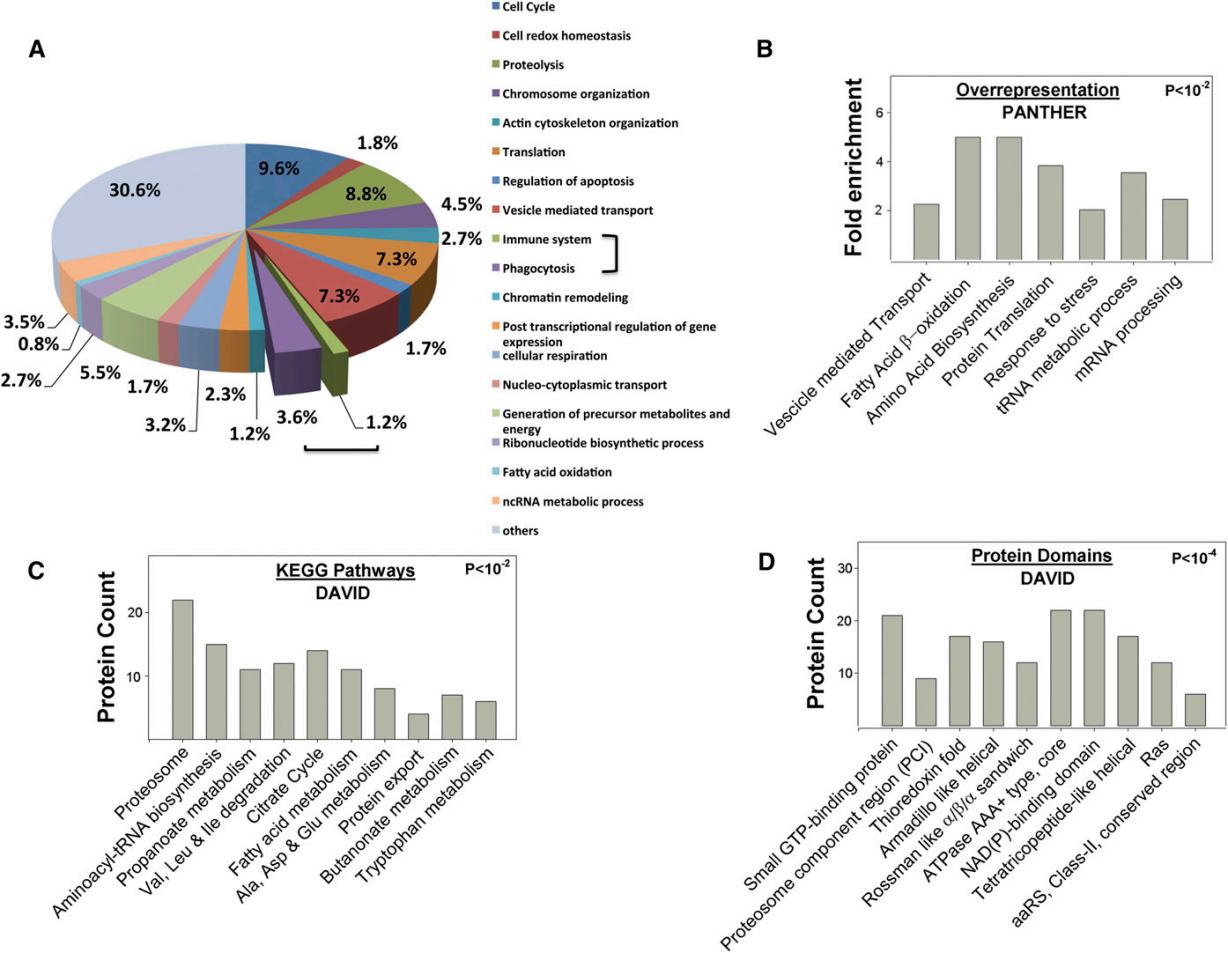
Analysis of the confident set. (A) A confident data set of 710 proteins, which is a subset of the 1619 unique proteins identified in the experiment, was used for Gene Ontology analysis using David Bioinformatics resource, where proteins are classified into various functional groups. Including phagocytic function, 4.8% of the proteins could be related to a function in immune response. (B) Fold enrichment, based on a statistical overrepresentation test, normalized to a standard *Drosophil*a data set, for few of the cellular processes with a *P* < 0.01. The PANTHER (Mi and Thomas 2009) resource is used to calculate the values, as described in *Materials and Methods*. The P values for the categories represented increases from left to right, with Vesicle transport having the value of 4.3 × 10^−5^. 621 proteins from the confident set are analyzed by the program (n = 621). (C) Number of proteins that show significant enrichment, normalized to a standard *Drosophila* data set, for functional pathways, as analyzed by the KEGG module of the DAVID bioinformatics resource (Huang da *et al.* 2009b). The *P* values are as calculated by the program (see *Materials and Methods*) and are ordered with increasing value from left to right, ranging from 4.0 × 10^−8^ (Proteasome) to 8.3 × 10^−2^ (Tryptophan metabolism), n = 249. (D) Number of proteins that show significant enrichment, normalized to a standard *Drosophila* data set, for protein domains, as analyzed by the DAVID bioinformatics resource (Huang da *et al.* 2009b). The *P* values are as calculated by DAVID (see *Materials and Methods*), range from 1.5 × 10^−7^ (Small GTP-binding proteins) to 9.8 × 10^−4^ (aaRS, class2 ), n = 614.

To obtain a confident set of proteins showing significant changes in response to LPS, as described in *Materials and Methods*, we chose proteins that are present in at least two or more biologic replicates with ITRAQ ratios ≥2.0 as significant hits. This list of 710 proteins comprises the “confident set” (Figure S1) of the LPS-induced SUMO-enriched proteome and is subsequently analyzed for global trends. Gene Ontology analysis using DAVID Bioinformatics resource (Huang da *et al.* 2009b) showed the identified proteins to be involved in diverse functions (Figure 2A). Based on a literature survey of immunity related genes, a significant fraction (∼4.8%) of the confident set could be directly linked to proteins known to be involved in immune function, inclusive of phagocytosis. The number is probably an underrepresentation as many of the other proteins identified in our screen may have yet undiscovered roles in immunity. The PANTHER enrichment analysis suggests (Figure 2B) that many biological processes has protein components regulated by SUMO. Vesicle mediated transport processes including endocytosis and exocytosis along with nuclear transport, response to stress were among processes significantly enriched. Proteins identified in this group include α-COP, β-COP, β′-COP, Sec23, KdelR, Peanut, Vps35, Rab1, Snap, and Eps-15. DAVID analysis was used to identify enriched protein domains and pathways (Figure 2, C and D). KEGG pathway (Ogata *et al.* 1998) analysis implicates metabolic pathways and members of proteasomal degradation pathway as SUMO targets. Enriched protein domains (Figure 2D) may indicate a common protein fold as a target for the SUMOylation machinery or may lead us to a protein domain family that is subject to SUMO mediated regulation in *Drosophila* and subsequently in eukaryotes. Our data predicts that among others, small GTP-binding proteins, proteasome component region folds, thioredoxin folds, and members of the ATPase AAA+ family are probably SUMO conjugated and may respond to immune stress in eukaryotes. A number of enzymatic/metabolic pathways involved in translation and degradation also are substantially enriched. For example, a large number of tRNA synthetases, Aats-Ala, Aats-Arg, Aats-Asn, Aats-Gln, Aats-Cys, Aats-EPRS, Aats-Gly, Aats-His, Aats-Ile, Aats-Lys, Aats-Tyr, Aats-Val, Aats-Ala, and Aats-Thr, were enriched in our experiment, with the last three excluded from the confident set having ITRAQ ratios <2.0. Our list also indicates enrichment of the SUMO conjugation machinery components including Uba2, a subunit of the activating enzyme, lwr/Ub9 and a *Drosophila* ligase Su(Var)2-10. SUMO (*smt3*) is also enriched but not listed in the 1619 set because it is also identified in the master control set. Similar kinds of enrichment patterns but with distinct protein signatures also are observed in previous proteomic studies in yeast and mammalian cells (Bruderer *et al.* 2011; Becker *et al.* 2013; Barysch *et al.* 2014; Hendriks *et al.* 2014; Tammsalu *et al.* 2014).

### Global changes in SUMOylation: the SUMO interactome

A significant proportion of the proteins identified by our proteomics experiment may be SUMOylated. Our affinity purification conditions (see *Materials and Methods*) allow strong protein−protein interactions to be maintained, leading to identification of strong interactors of SUMOylated proteins. Because SUMOylation can act by regulating the interactions of the substrate protein with other macromolecules, such information is useful. SUMOylation of a substrate can create a binding site and/or modulate conformation leading to the enhancement or decrease in binding affinity. SIMs have been discovered, pointing to a significant role for SUMO in modulating protein−protein interactions, especially in relation to large protein complexes. Many previously published studies have proposed that multiple proteins are SUMOylated in a single functional complex. To analyze our list in terms of global protein interactions, we turned to a comprehensive study on protein−protein interactions in S2 cells, the same system we used for our studies. Guruharsha *et al.* (2011) have combined high-throughput affinity pulldowns of nearly 5000 individual proteins and used MS to identify a large component of the global protein:protein interaction network (DPiM). 

**Figure 3:**
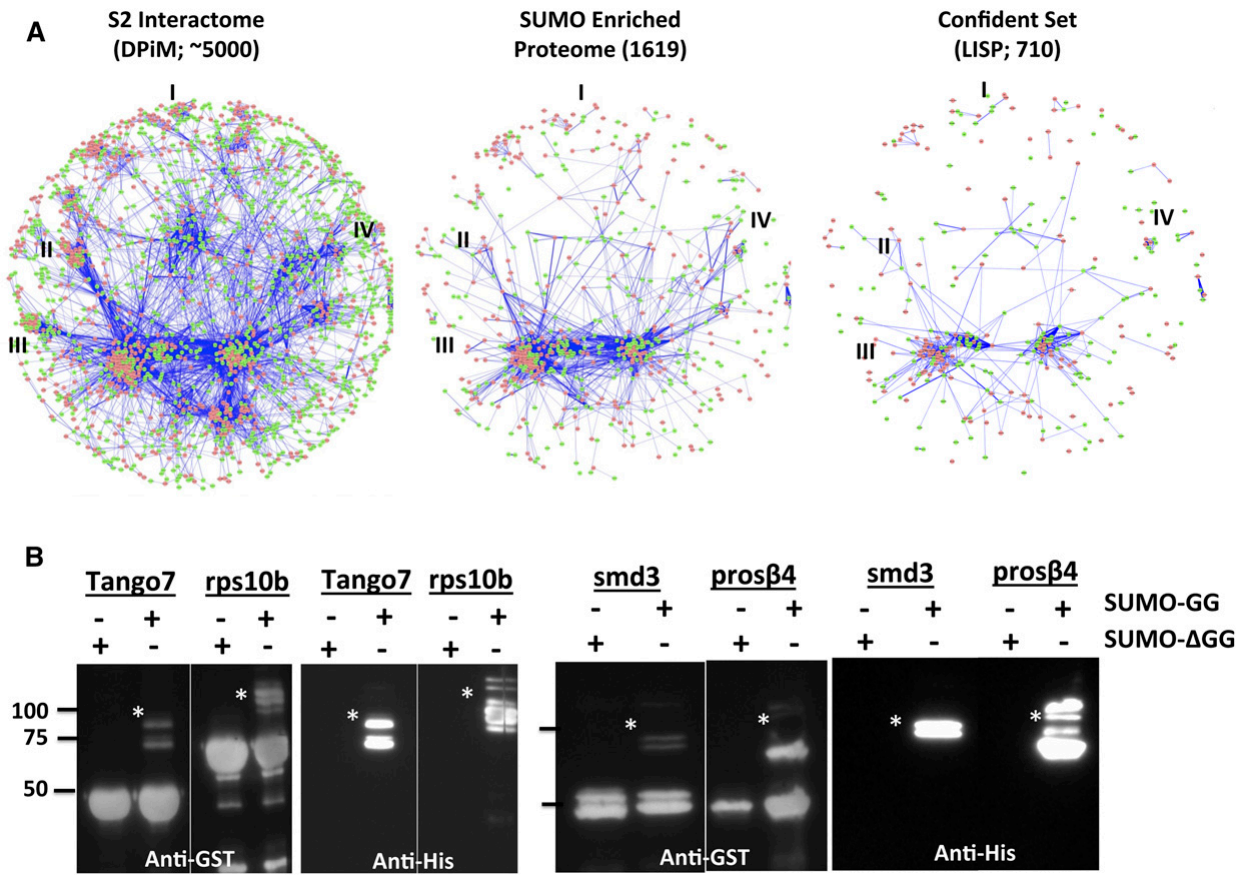
The small ubiquitin-like modifier (SUMO) proteome enriches specific protein complexes. (A) Cytoscape representation of a molecular interaction networks for S2 cells, based on data from Guruharsha *et al.* (2011). The figure on the left represents an interaction map of 4500 proteins (DPIM), as discovered by a large-scale affinity purification experiment. The figure in the middle represents a combined SUMO-enriched proteome of 1619 proteins we have generated and mapped onto the DPIM map. Many complexes such as the Histone Acetyl Transferase Complex (I), Mediator Complex (II), the SNARE/Syntaxin Cluster (III), and the Arp/Arc complex (IV) are underrepresented in the SUMO-enriched network. The figure on the right is a map of the confident set, with 710 proteins. (B) Validation of a few proteins that are part of large protein complexes identified in S2 cells (Guruharsha *et al.* 2011) and listed in Table 1. The system used for validation is the *in bact*o ‘Q’ system (Nie *et al.* 2009). Proteins to be validated are coexpressed as GST fusions in bacteria along with 6XHis-SUMO-GG (or 6XHis-SUMO-ΔGG) and E1 and E2 enzymes. In the Western blots shown, SUMOylated proteins (marked with a *) can be identified by the presence of a weak, higher molecular weight band (15 kDa or more) in the anti-GST blots that also cross-reacts with the Anti-His antibody. Proteins shown to be SUMOylated include Tango 7 (part of the eIF3 complex), rps10b (ribosome small subunit), smD3 (spliceosome) and prosß4 (proteasome).

Because the SUMO-enriched proteome would represent a subset of proteins in S2 cells that is biased toward SUMOylation or SUMO interaction in the immune response, we mapped our set of 710 proteins that represent ∼14% of the proteins the total DPiM network of 5000 proteins (Figure 3A) onto the DPiM network. The comparison is not absolute because the methods used to generate the interactors involved are different, but the analysis leads to interesting findings. First, many of the major clusters/complexes shown in the wild-type DPiM are missing in the SUMO-interactome (Figure 3A; Table 1). The underrepresented complexes include the Mediator, SNARE/Syntaxin, Ribosomal, Augmin, and Arp/Arc protein complexes (Figure 3A). Other known complexes, such as the Tango complex, eIF3, Proteasome as well as the Multi Acyl tRNA synthetase (MARS) complex, are, however well represented (Table 1). Our analysis suggests that specific protein complexes respond to immune stress and have components that are SUMOylated. For example, the *Drosophila* glutamyl-prolyl tRNA synthetase (EPRS) is SUMOylated (Smith *et al.* 2004), and its human ortholog is part of the GAIT and MARS complex, with roles in immune signaling (Jia *et al.* 2008), but its role and existence in *Drosophila* immunity is yet to be elucidated.

**Table 1:** Members of protein complexes that are enriched and not enriched in the SUMO proteome

Complex (Number of Members)	Proteins Listed in SUMO-Enriched Proteome	Not Identified in SUMO-Enriched Proteome
MARS complex (13)	Aats-arg, Aats-glupro, Aats-lys, Aats-glu, Aats-ile, Aats-leu, Aats-asp, Aats-Glu, CG8235, CG15100, *CG12304*, *CG33123* (12)	Aats-met (1)
RNA processing-exosome (RNase complex; 9)	Rrp6, Rrp4, RRp40. Rrp46, *Dis3*, *Rrp42*, (6)	Ski6, Mtr3, Csl4 (3)
SNAP-SNARE complex (31)	Snap, Nsf2 (2)	Syx16, usnp,Syx1A, Slh, Use1, gammaSnap, Slh, membrin, Snap25,Sec22, Syx8, Snap24, Syx5, CG1599, Ykt6, CG2023, Syx13, Syx4, Koko, Syx18, Vti1,Syb, Syx, Bet1,CG6208, Rme-8, AttD, Syx17, n-syb (29)
Proteasome complex (50)	Rpn12, Pros54, Rpn9, Rpn7, CG13349, Prosbeta2, Prosalpha7, Rpn5, Mov34, Pros45, Prosbeta5, Pros29, Pros35, Rpn1, Pros26.4, Prosbeta3, Prosbeta7, Rpn2, Rpt3, Rpn6, Tbp-1, Rpn1, CG17331, Pros26, Prosalpha5, Rpt4, REG, Ufd1-like, Rpt1, *Uch-L3*, *Rpn3* (31)	CG11885, CG2036, Prosbeta4R2, Prosbeta2R1, pomp, Prosalpha6T, Prosbeta4R1, Prosbeta1, pros28, Pros25, Prosalpha1, rpr, CG12321, CG2046, CG13319, GNBP2, CG11885, CG3812, CG9588 (19)
eIF3 complex (17)	Tango7, eIF3-p66, Int6, eIF3-s8, eIF3-S10, eIF3-S9, eIF3-ga, eIF3-gb, CG10306, CG5642, *Trip1*, *CG9769*, *eIF3-p40*, CG5651 (14)	Adam, CG4810, CG8335 (3)
Escrt complexes (21)	RAB11, RAB7, TSG101, VPS4, RAB8, RAB4, VPS28 (8)	RAB5, HRS, VPS23, VPS37, VPS36, VPS25, VPS20, VPS60,VPS46, VPS24, VPS2, VTA1, SNF7, RAB35, (14)
Mitochondrial ribosomal protein complex (large subunit) (47)	mRpL2, mRpL17, *mRpL19*, *mRpL41* (4)	mRpL1,mRpL3,mRpL4,mRpL9,mRpL10, mRpL11,mRpL12, mRpL13,mRpL14, mRpL15,mRpL16, mRpL18, mRpL20, mRpL21, mRpL22, mRpL23,mRpL24, mRpL27, mRpL28, mRpL30, mRpL32, mRpL33, mRpL34, mRpL35, mRpL36, mRpL37, mRpL38, mRpL39, mRpL40, mRpL42, mRpL43, mRpL44, mRpL45, mRpL46, mRpL47/Rlc1, mRpL48,mRpL49,mRpL50,mRpL51, mRpL52, mRpL53,mRpL54, mRpL55 (43)

The enrichment of proteins in the SUMO-enriched proteome suggest that SUMOylation may play a role in regulating complex formation or may modify activity of the complex. Proteins that are found in the 1619 unique set, but not in the 710 member confident set, are italicized. Data for physical SUMO modification of a few proteins in protein complexes are shown in Figure 3B. SUMO, small ubiquitin-like modifier.

### Validation of targets

To gain confidence on the ability of the screen to identify genuine SUMO substrates, we needed to confirm that our confident set contains *bona fide* SUMOylated targets. Literature from the SUMOylation field clearly indicates that only a small proportion of any protein in the cell is SUMOylated at any given time, indicating that a SUMOylated species may not be detected by our methods, even after enrichment of substrate. To maximize our chances for demonstrating SUMO modification, we used *in-bacto* system (see *Materials and Methods*) developed by the Courey Lab (Nie *et al.* 2009). This system has the advantage that bacteria lack a SUMO deconjugase and that substrate proteins can be expressed in milligram amounts allowing detection of the rarer SUMOylated species. Use of *in bacto* SUMOylation enhances our ability to demonstrate physical SUMOylation; it does, however, not guarantee it. The system works for proteins that do not require an E3 ligase and are soluble when expressed in bacteria. We have validated a few genes that are in proteins complexes (Table 1). Figure 3B shows that Tango7 (IF3 Complex), Prosβ4 (Proteasome Complex), SmD3 (Splicing Complex), and Rps10b (Ribosome, small subunit) are SUMOylated using the *in bacto* system. Also SUMOylated is Aats-Arg (data not shown), a member of the MARS complex. Many of these complexes are thus possible components of the response to infection by the cell. To further bolster our data, many proteins in Table 1 have been shown to be SUMO targets by other researchers (Hendriks *et al.* 2014).

### Discovery of novel targets of the immune response

The larger dataset of 1619 proteins was divided into two subsets, namely the biased and the unbiased set. For the *unbiased* set we chose 50 proteins with the greatest ITRAQ ratios and we predict that these have undiscovered roles in immune signaling (Table 2). This is based on the logic that a significant increase in SUMOylation indicates involvement of these proteins indirectly or directly in the broad immune response. Nine of the proteins listed in Table 2 have already been shown to be SUMOylated, including one, SmD3, discovered in this study. For the *biased* approach, we used a literature survey to identify 50 proteins that could be implicated, directly or indirectly to the immune response. The process followed to list these proteins included looking at databases and scanning the literature on innate immunity. Table 3 lists these proteins, their molecular function, and their current known SUMOylation status. Since data for our screen were first collected in 2012, orthologs of many proteins discovered in our proteomic screen have now been show to be SUMOylated in mammalian cells (Hendriks *et al.* 2014). Many targets in our list that are not yet validated SUMO targets have putative SUMOylation motifs and/or SUMO interacting motifs. For molecules involved in immune signaling, previous studies have shown physical evidence of SUMOylation of *Drosophila* include DL, STAT92E (Bhaskar *et al.* 2002; Gronholm *et al.* 2010), Ras85D (Nie), and Ird5 (Fukuyama *et al.* 2013). In mammals, orthologs of Jra and Kay (Bossis *et al.* 2005), AGO2 (Sahin *et al.* 2014), Suv(var)2-10, Rm62, and Pvr have been shown to be SUMOylated (Table 3).

**Table 2:** Fifty proteins from the confident set, with greatest ITRAQ ratios, arranged in descending order of ratio

Protein	Molecular Function	Lysine Residue(s) Predicted to be SUMOylated	No. of Predicted SUMO Binding Motifs	Previously Demonstrated to be SUMOylated	ITRAQ Ratio
SmF	mRNA splicing	120, 341	2		4.346
SmG	mRNA splicing	0	0		3.886
Mtor	Ribonucleoprotein complex binding	19, 63, 101, 138, 155, 272, 753, 762, 859, 878, 939, 988, 1073, 1092, 1221, 1226, 1518, 1549, 1551	1		3.641
SmB	mRNA splicing	194, 221, 509, 585, 757, 991	3		3.619
CG42383 (eyc)	Photoreceptor development	6	2		3.427
PPP4R2r	protein phosphatase regulator activity	214, 238, 328, 334, 339, 352, 380, 394, 419, 426, 591	0		3.375
Ena	SH3 domain binding	229, 663, 805, 820, 828	0		3.370
CG8683 (mon2)	Cytoskeletal regulatory protein binding	82, 103, 124, 512, 1325, 1512	4		3.318
CG32176	Unknown	22, 31, 189, 266, 462, 543	0		3.299
snRNP-U1-70K	mRNA splicing	80, 117, 362, 374, 393, 403	0		3.242
SmD2	Poly-A RNA binding	7	2	HeLa (Hendriks *et al.* 2014)	3.238
Lds	DNA-dependent ATPase activity	280, 285, 389, 456, 492, 546, 565, 696, 910, 1033, 1056	2	HeLa (Hendriks *et al.* 2014)	3.220
Prosbeta3	Threonine-type endopeptidase activity	155	0	HeLa (Becker *et al.* 2013)	3.178
Tango9	Nucleotide-sugar transporter	36	0		3.172
Adk3	Adenylate kinase activity	62, 148, 163, 188	1		3.121
Rbp1	RNA binding	0	0		3.108
Polo	Protein serine/threonine kinase activity	4, 150, 456	1		3.085
Lwr	Ubc9; SUMO E2 conjugase	14, 65	0	HeLa (Hendriks *et al.* 2014); HeLa (Impens *et al.* 2014)	3.068
CG3939	Unknown	0	1		3.064
SmD1	Poly-A RNA binding	0	0	HeLa (Hendriks *et al.* 2014)	3.038
SH3PX1	Phosphatidylinositol-4,5-bisphosphate binding	227, 231, 328, 455, 528	0		3.019
CG12567	Thiamine diphosphokinase activity	7, 45, 115, 123, 169	0		3.017
Ced-12	Cell motility	220, 250, 360, 693	1		3.009
Msp-300	Actin binding;	69 predicted sites	10		3.002
RpLP2	Translation regulation	92	0		2.977
Prod	Chromatin binding	4, 123, 321	0		2.956
SmD3	Poly(A)-binding protein	0	0	*In bacto*, this Study; HeLa (Hendriks *et al.* 2014)	2.944
CG5728	mRNA binding	103, 228, 540, 544, 588, 679, 701, 718, 995, 1078, 1104, 1112, 1163, 1225, 1229, 1245, 1366, 1384, 1417	3		2.939
CG40045	Ubiquitin-protein transferase activity	101	0		2.926
Fis1	Mitochondrial fission	20, 36, 114	0		2.925
Nmdmc	Tetrahydrofolate dehydrogenase	14, 29, 159, 189, 251	2		2.921
Map60	Microtubule binding	84, 88, 169, 297, 357, 421	0		2.909
CG5214	Dihydrolipoyllysine-residue Succinyltransferase activity	94, 422, 426	0		2.902
CG3402	Wnt signaling	0	0		2.902
CG7945	Unfolded protein binding	214	0		2.901
Nct	Photoreceptor development	0	3		2.899
CG11505	Nucleotide binding	327, 355, 1339	1		2.896
Ge-1	mRNA processing	132, 136, 216, 231, 307, 355, 394, 480, 571, 630, 897, 951, 1270, 1328	3		2.895
Rl	Jun kinase activity	92, 164, 177	1		2.894
HP1b	Chromatin binding	224	0		2.880
Pof	mRNA binding	92, 201, 247, 320, 346, 397, 421	0		2.879
CG12065	Nucleoside phosphorylase	102, 341	2		2.862
CG17544	acyl-CoA dehydrogenase activity;	0	0		2.861
Cortactin	Proline-rich region binding	230, 246, 348, 354, 456	0	HeLa (Hendriks *et al.* 2014);	2.835
				HeLa (Impens *et al.* 2014)	
SrpRβ	GTPase binding	0	1		2.820
Ctf4	DNA endoreduplication	120, 147	1		2.818
CG2034	Unknown	212	0		2.815
Rtnl1	ER organization	9, 81, 87, 272, 356, 363, 403	0		2.809
eIF4AIII	Translation initiation factor activity	34, 230, 252, 370	1	HeLa (Hendriks *et al.* 2014)	2.795
CG1518	Oligosaccharyl transferase activity	464, 576	1	HeLa (Hendriks *et al.* 2014)	2.776

These proteins represent potential SUMO targets in S2 cells that respond to LPS. Listed are Proteins names, their molecular function, potential SUMOylated lysines as well as SIMs, based on the JASSA algorithm (Beauclair *et al.* 2015). The ITRAQ ratio is an average of values observed in biological replicates. SUMO, SUMO, small ubiquitin-like modifier; ITRAQ, isobaric tag for relative and absolute quantitation; mRNA, messenger RNA; ER, endoplasmic reticulum; SIM, SUMO interaction motif.

**Table 3:** Representative immunity-related hits from the ITRAQ data set, sorted on ITRAQ ratio

Protein	Molecular Function	Lysine Residues Predicted to be SUMOylated	Predicted SUMO Binding Motifs (SIM)	Demonstrated to be SUMOylated	ITRAQ Ratio
*Basket*	*JNK signaling*	*73*, *151*, *158*, *316*	*0*		*2.96*
Rolled	MAP kinase pathway	92, 164, 177	96-99		2.89
Su(var)2-10	DEAD/H-box binding	294, 406, 439, 490, 493	54-57	HeLa (Hendriks *et al.* 2014)	2.73
Kay	JNK transcription factor	520, 533	0	HeLa (Hendriks *et al.* 2014)	2.72
Cdc42	GTP binding protein	150	0	*In bacto*, this study	2.70
CG14207 (HspB8)	Endogenous TLR ligand; Heat Shock Protein	93, 117, 123	0		2.66
AGO2	Endonuclease involved in siRNA mediated silencing	3, 438, 470, 813, 948, 1048, 1193	887-890, 1071-1074	HeLa (Sahin *et al.* 2014)	2.65
Cpa	Negative regulation of JNK pathway; Actin regulator	39, 95, 189	0	Not SUMOylated, *In bacto*, this study	2.64
*Sec5*	Promotes vesicular trafficking	67, 139, 179, 226, 309, 457, 742	*0*		2.63
CG7593	N-Acetyl transferase, Role in Phagocytosis.	43, 49, 55, 180	0		2.61
Ras85D	Small GTPase; Hindgut Immunity.	5, 170	0	*In bacto*, (Nie *et al.* 2009)	2.60
*TRAM*	*Involved in phagocytosis*	*316*, *320*, *324*, *328*	*213-216*		*2.46*
Snap	Vesicular transport protein	7, 18, 151, 240	*0*		2.40
*Hrs*	*Involved in endocytosis*; *Escrt0 complex*	*263*, *747*	*0*	*-*	*2.36*
Stat92E	Transcription factor; JAK-STAT pathway	24, 187, 241, 446, 455, 685	84-87, 480-483, 481-484	*Drosophila*, (Gronholm *et al.* 2010)	2.33
Mask	Structural component of cytoskeleton	91, 615, 2345, 2786, 2873, 3876	997-1000, 1030-33, 2361-64, 2395-2398, 2598-2601	−	2.30
P38b	MAP kinase pathway	10, 155	83-86	HeLa (Hendriks *et al.* 2014)	2.29
*Mbo*	Nuclear Transport factor	118, 240, 371, 477, 557	*0*	−	2.26
TepIV	Complement like proteins in anti-microbial response	25, 29, 156, 172, 187, 251, 387, 619, 625, 635, 1137, 1190, 1287, 1297, 1331, 1352, 1437	1082-1085, 1427-1430		2.25
Rho1	GTP-binding protein required in cell shape changes	135	111-114		2.24
Jra	JNK transcription factor	29, 190, 214, 248	0	*In bacto*, this study; HeLa (Hendriks *et al.* 2014)	2.23
Psidin	Involved in phagocytosis	76, 131, 192, 236, 697, 853, 909	762-765		2.23
Pvr	Transmembrane receptor protein tyrosine kinase	67, 129, 159, 258, 409, 599, 602, 731, 740, 750, 883, 887, 920, 944, 1092, 1451	247-250	HeLa (Hendriks *et al.* 2014)	2.22
14-3-3ε	Involved in signaling and protein transport	73, 78, 118, 125	0	*In bacto*, this study	2.18
Rab11	Rab family GTPase required in endocytic recycling	140-201	14-17	*In bacto*, this study	2.18
Mus209	DNA polymerase processivity factor activity	117, 254	0		2.17
Hop	protein tyrosine kinase activity	618, 698, 724, 754, 839	1147-1150		2.17
Hel89B	ATP-dependent DNA helicase activity	84, 341, 596, 600, 606, 615, 624, 964, 1094, 1142, 1241, 1308, 1313, 1584, 1832	0	—	2.15
*Colt*	*Transport protein involved in phagocytosis*	*12*, *69*, *112*, *154*	*0*	*-*	*2.10*
*Pvf2*	*Vascular endothelial growth factor receptor*	*282*	*0*		*2.09*
Rm62	DEAD-box RNA helicase	374	362-365	HeLa (Hendriks *et al.* 2014)	2.03
*Trip1*	*Translation initiation factor activity*	*33*, *149*, *229*, *266*	*124-127*	*HeLa (**Becker et al. 2013**)*	*1.99*
*Ntf-2*	*Nuclear transport factor*	*-*	*0*		*1.96*
*Epsilon-Cop*	*Vesicular transport protein*	*274*	*0*		*1.95*
*Drk*	*SH3/SH2 adaptor protein in sevenless signaling*	*6*, *146*	*178-181*	*-*	*1.88*
*Rac1*	*GTPase involved in actin organization*; *JNK signaling*	*49*, *133*	*109-112*		*1.83*
*Ral a*	*Ras-like GTPase*	*112*	*0*		*1.77*
*Dos*	*Adaptor protein in Sevenless signaling*; *Phagocytosis*	*276*, *580*, *718*, *781*	*0*	*-*	*1.64*
*TM9SF4*	*Protein transporter*	*41*, *45*, *76*, *211*, *248*, *628*	*0*		*1.62*
*Casp*	*Negative regulator of IMD/NFκ-B signaling*	*263*, *414*, *436*, *484*, *551*, *660*	*213-217*	*In bacto*, *in S2 cells*; *This Study*	*1.35*
*Imd*	*Involved in signal transduction of immune response*; *IMD/NFκ-B pathway*	*153*	*0*		*1.35*

**Figure 4:**
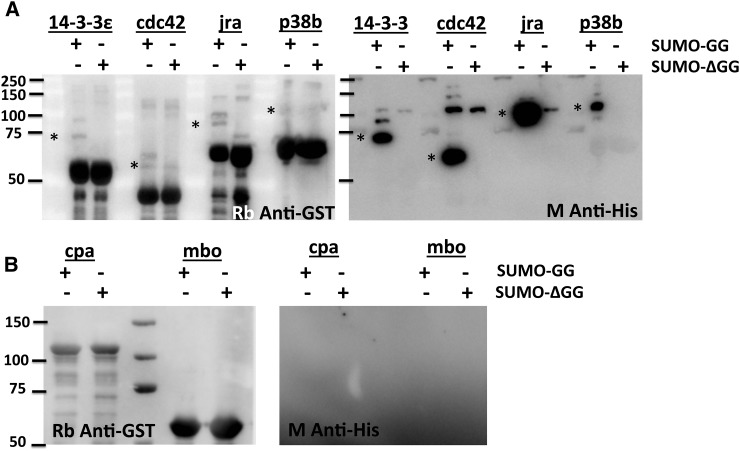
SUMOylated proteins involved in the immune response. Western blots of a subset of immune-related proteins identified in this study, as listed in Table 3. Twelve proteins were tested, using *in bacto* SUMOylation, with seven showing SUMOylation and five not showing SUMOylation. (A) A representative set of proteins that are SUMOylated using the *in bacto* assay are shown in the figure. SUMOylated proteins (marked with a *) can be identified by the presence of a weak, higher molecular weight band (15 kDa or more) in the anti-GST blots that also cross-react with the Anti-His antibody. (B) A representative set of proteins that were not SUMOylated using the *in bacto* assay.

These proteins represent potential SUMO targets in involved in the cellular response to LPS. Listed are Proteins names, their molecular function, predicted SUMOylated lysines as well as SIMs, based on the JASSA algorithm (Beauclair *et al.* 2015). The ITRAQ ratio is an average of values observed in biological replicates. The italicized proteins are not part of the confident set but are part of the larger, 1619 member unique set. Proteins shown to be SUMOylated in this and earlier studies are indicated in column 5. ITRAQ, isobaric tag for relative and absolute quantitation; SUMO, SUMO, small ubiquitin-like modifier; SIM, SUMO interaction motif; LPS, lipopolysaccharide;

For validating targets in our immune list, we tested 12 proteins involved in immune signaling for SUMOylation and could demonstrate that seven of the proteins, namely 14-3-3ε, Cdc42, Jra, Kay, p38b, Casp, and Rab11, were SUMOylated *in bacto*. Representative examples are pictured in Figure 4A. Many of the targets could not be shown to be SUMOylated *in bacto*, and they include Cpa, Mbo, Snap, basket, Hrs, with representative examples pictured in Figure 4B. The demonstration of SUMOylation of some targets from our screen is encouraging and leads us to believe that we will be able to demonstrate SUMOylation of many more targets from Table 3 as well as from the confident set. The identification of SUMOylation targets is of course only a first step for a detailed analysis of the effect of a SUMO tag on each protein identified. For each protein, mutants at single or multiple lysine sites that block SUMOylation have to be identified and generated. This can be done in a reasonable time frame using the *in bacto* system, where we can mutagenize genes and test lack of SUMOylation in mutant (Lys→Arg) constructs. Then, the effect of each mutant on immune function can then be explored in cultured cells and *in vivo*.

### Casp is SUMOylated both *in bacto* and cells in culture

**Figure 5:**
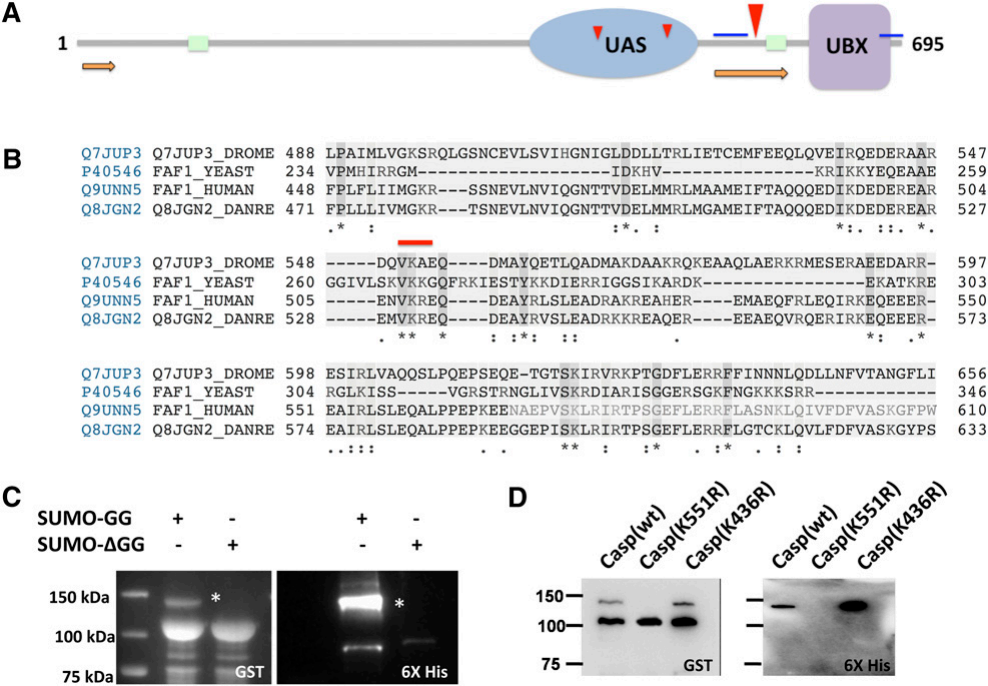
Casp/FAF-1 SUMOylation. (A) Domain structure of *Drosophila* FAF1. The UAS and UBX domains are marked with potential SUMOylated sites (red arrowheads). The largest arrowhead (K551) has a consensus small ubiquitin-like modifier (SUMO) acceptor site sequence. The gray line indicates disordered regions, green boxes are low-complexity regions, whereas the orange bars indicate regions with predicted helical structure. (B) Alignment of FAF1 proteins from *Drosophila*, yeast, human, and zebrafish. The figure shows a section of the aligned sequences that contains the predicted SUMO acceptor site (VK^551^AE) and is conserved from yeast to man. (C) Casp is SUMOylated when tested using the *in bacto* system. An additional band (*) is seen for GST-SUMO in the presence of activated SUMO (SUMO-GG) but not when SUMO-ΔGG, an inactive form of SUMO that cannot conjugate is used in the SUMOylation assay. (D) K551 is the SUMO acceptor site in Casp. A Casp(K551R) mutation leads to a loss of the SUMO band while a Casp(K436R) mutation does not affect SUMOylaton. The assay is carried out using *in bacto* SUMOylation.

Of the *bona fide* targets SUMO targets discovered in our *in bacto* screen, we chose Casp as a target for validation in S2 cells in culture. Casp was identified in a genetic screen (Kim *et al.* 2006) for *Drosophila* mutants with hyperactivated immune response. A homolog of mammalian Fas-associating factor 1 (Chu *et al.* 1995) (FAF1; Figure 5A) negatively regulates IMD/NF-κB−mediated immune response (Park *et al.* 2004; Kim *et al.* 2006). FAF1 is an important cellular protein with adaptor roles in neurogenesis (Cheng *et al.* 2011; Sul *et al.* 2013), protein turnover (Lee *et al.* 2013), and tumorigenesis (Menges *et al.* 2009; Lee *et al.* 2012). Casp, like other FAF1 members, contains a UAS domain (IPR006577), with an unknown functional significance and an Ubx domain (IPR001012), which is found in proteins involved in ubiquitin regulatory pathways (Menges *et al.* 2009). SUMO prediction software SUMOsp (Ren *et al.* 2009) predicted two weak consensus sites for SUMOylation; K436 and K484, and one strong consensus site (K551; Figure 5A; marked with red arrowheads). Amino acid alignment of the *Drosophila* Casp with its homologs in humans, zebrafish, and mice showed only one of these SUMO sites, namely K551 conserved across species (Figure 5B). Mutation of lysine K551 to arginine showed loss of SUMO modified form of the protein confirming that K551 was the site of SUMOylation (Figure 5, C and D). Mutations of K436 (data not shown) or K484 (Figure 5D), in contrast, did not affect SUMO modification. 

**Figure 6:**
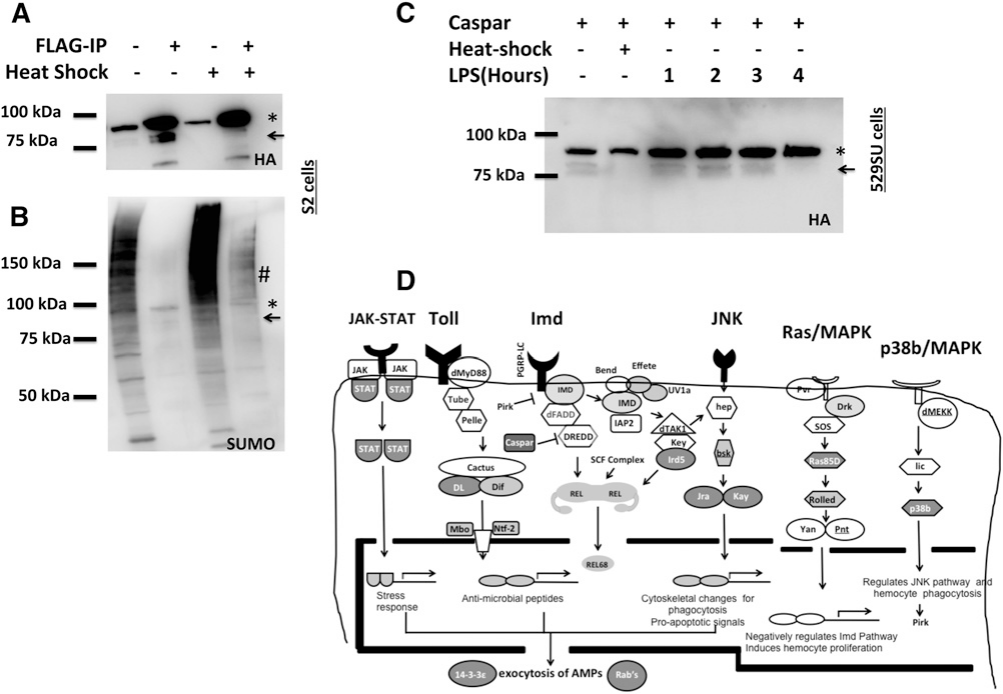
Casp is SUMOylated in cultured cells. (A) A Western blot of cellular lysates after transient expression of Casp-HA-FLAG in S2 cells shows two bands (Lane 1 and 2), a ∼80-kDa minor band (→), and a ∼95-kDa major band (*). Both bands react to anti-HA antibody and can be concentrated by anti-FLAG affinity. On heat shock (Lane 3 and 4), the lower band is not pulled down by FLAG-agarose, suggesting a transition to a SUMOylated state when cells are stressed. (B) When the blot shown in Figure 6A is probed with a SUMO antibody, it shows a characteristic ladder (Lane 1 and 4) of SUMOylated species. In the anti-FLAG affinity samples, the 95-kDa band cross reacts with the antibody, suggesting that the higher band corresponds to a SUMOylated version of Casp. As expected, a heat shock response leads to an accumulation of higher molecular weight small ubiquitin-like modifier (SUMO) species is also seen (Lane 3). The Casp affinity purification also seems to bring down other SUMOylated species (#). (C) A Western Blot, probed with anti-HA antibody showing the response of cells to LPS. Casp-HA-FLAG is transiently expressed in 529SU cells and the immune response initiated by the addition of 10 μg/mL lipopolysaccharide (LPS). The major Casp band corresponds to a SUMOylated species (95 kDa), with the non-SUMOylated species (80 kDa, →) decreasing with increasing time post-LPS treatment. (D) SUMOylated proteins can act as dynamic regulation points in immune signaling. This figure summarizes the current status of proteins regulated by SUMO in *Drosophila* immune response. The Tl, immune-deficient (IMD), Jun kinase (JNK), mitogen-activated protein kinase (MAPK), and Janus kinase/signal transducers and activators of transcription (JAK-STAT) pathways appear to have at least one control point for SUMO-mediated regulation. Proteins labeled in white, within dark gray boxes, have been demonstrated to be SUMOylated in this and previous studies. Proteins labeled in black, in light gray boxes, are potential targets that need further validation.

Next we tested whether Casp was SUMOylated in S2 cells. Demonstration of physical SUMOylation in cultured cells and *in vivo* is a challenging task, primarily because of the small percentage of SUMOylated species, compared with the total species in the cell. We expressed a Casp-HA-FLAG construct in both 529SU and S2 cells and looked for physical evidence of a SUMOylated species. We found, over multiple experiments, that both the SUMOylated and non-SUMOylated form of Casp exist in S2/529SU cells with stress causing a transition to the SUMOylated state. Western blots of S2 cells expressing Casp-HA-FLAG, when probed with anti-HA antibody, show a weak band at ∼80 kDa (arrowhead), which is enriched on FLAG-IP and which can be converted to a greater molecular weight form (stronger band; ∼95 kDa) by heat shock stress (Figure 6A). The 95-kDa band but not the same 80 kDa band cross-reacts with the anti-SUMO antibody (Figure 6B) for the same blot, suggesting that the 95-kDa band is the SUMOylated form of Casp. Heat shock thus causes a conversion of the non-SUMOylated 80-kDa Casp form (76 kDa based on primary sequence) to a SUMOylated form with additional SUMOylated species (#) being copurified along with Casp during affinity purification. Because we originally identified Casp in an LPS-induced proteomics experiment in 529SU cells, we also looked at the effect of LPS addition to SUMOylation of Casp. In 529SU cell lysates, as shown in Figure 6C, Casp is primarily in the 95-kDa SUMOylated form (marked by *) with addition of LPS leading to a decrease in the intensity of non-SUMOylated Casp (80 kDa, marked by an arrow) as it transits to a SUMOylated state (4 hr LPS), similar to the transition seen when cells are heat shocked (Figure 6A, Lane 4).

## Discussion

SUMOylation is an important PTM in the cell. Ideally, as a starting point in understanding global functional roles for SUMOylation in the cell, one would like to have a comprehensive list of cellular proteins that are SUMOylated. With increasing number of technical advances in MS and the ability to purify SUMOylated species using protein tags or antibodies, this goal is increasingly within our reach. Extensive lists of SUMOylated proteins have been made, both by mapping SUMO fragments conjugated to substrate targets directly by MS as well as by biochemical purification of SUMOylated proteins by various methods (Wohlschlegel *et al.* 2004; Zhou *et al.* 2005a; Vertegaal *et al.* 2006; Golebiowski *et al.* 2009; Hendriks *et al.* 2014) and subsequent identification of peptides by MS. A single SUMO proteome data set for *Drosophila* exists for 0- to 3-hr embryos (Nie *et al.* 2009). One fact that is established from these studies is that the SUMOylation state of a protein, namely a list of SUMOylated proteins, is dynamic and may change based on cell type (Nie *et al.* 2009; Becker *et al.* 2013), developmental stages (Nie *et al.* 2009), as well as with change in cellular stimuli (Golebiowski *et al.* 2009; Hendriks *et al.* 2014). The most comprehensive data have come from experiments that show that cells, when exposed to physical or chemical stress (Golebiowski *et al.* 2009; Tatham *et al.* 2011; Becker *et al.* 2013; Hendriks *et al.* 2014), show significant differences in the identity of proteins that are SUMOylated. For example, in the case of mammalian cells, studies have indicated that heat shock changes the SUMO proteome, with up to >50% new proteins SUMOylated after cells are heat shocked (Golebiowski *et al.* 2009; Hendriks *et al.* 2014). The SUMO proteome thus changes with cell types and stimuli and information about these changes will help researchers understand the importance of SUMOylation in different regulating cellular processes.

SUMO modification has been suggested as an important regulator of the immune response (Bhaskar *et al.* 2002; Chiu *et al.* 2005; Huang *et al.* 2005; Pascual *et al.* 2005; Paddibhatla *et al.* 2010), with important players in immune signaling pathways being SUMOylated, interacting with SUMOylated species or being modified by elements of the SUMO cycle (Desterro *et al.* 1998; Bhaskar *et al.* 2002; Huang *et al.* 2003; Kubota *et al.* 2008; Gronholm *et al.* 2010; Fukuyama *et al.* 2013). Our study is one of the first studies that attempt to collect a global picture of cellular changes in response to immune stress and identifies cellular processes and specific SUMO substrates that are modified by SUMO. We have looked at the change in SUMOylation state in *Drosophila* S2 cells after exposing cells to LPS. *Drosophila* SUMO, like mammalian SUMO-2 (Hendriks *et al.* 2014), is not amenable for proteomic identification when conjugated to the substrate due to the large size of its C-terminal tryptic fragment. Hence, our methodology involves enriching the SUMO proteome from cellular lysates before and after activating the immune response. Validation of physical SUMOylation and discovery of the SUMOylation site is downstream of the MS experiment by using *in bacto* SUMOylation.

In flies, the most dramatic effect of the initiation of infection is the up or down regulation of about 400 defense genes (De Gregorio *et al.* 2001; Irving *et al.* 2001; Johansson *et al.* 2005), defining the immune transcriptome. A comparison of the two data sets, the 1619-member SUMO-enriched S2 cell proteome, with the Immune transcriptome [(De Gregorio *et al.* 2001); http://lemaitrelab.epfl.ch/page-7767-en.html] indicates a <2% overlap. This finding is an interesting one with the lack of overlap highlighting the distinct spatiotemporal roles for dynamic SUMOylated proteins in the global immune response. The ∼400 defense proteins, which are expressed at the time point of MS data collection, namely 3 hr, are thus not represented in the SUMO-enriched immune proteome. The *Drosophila* SUMO immune proteome instead shows enrichment of cellular processes (Figure 2, A and B) such as RNA metabolism, nuclear transport, tRNA amino acylation, vesicular transport, and endocytosis/exocytosis. Similar but distinct processes have been implicated as being modified by SUMO in previous studies. Our study suggests major roles for SUMO-mediated regulation associated with translation, as well as in proteasomal degradation. In addition, protein domains such as small GTP binding proteins, nucleic acid binding (OB fold, alpha beta plait) fold, proteasome component region folds, and Thioredoxin fold proteins appear to be major targets of the SUMO machinery. As described in the *Results* section, one or many members of important cellular protein complexes, such as MARS, eIF3, MCM(2-7), ribosome small subunit, and the RNAse-Exosome complexes, appear to respond to LPS treatment, with one or many components SUMOylated, leading to a possible regulation of these functional complexes by SUMOylation.

Our data, summarized in Figure 6D, suggest that in TL/NF-κB signaling, in addition to DL (Bhaskar *et al.* 2002), nuclear transport factor-2 (*i.e.*, Ntf-2) is modified by SUMO, although the latter target have yet to been validated. Previous studies (Anjum *et al.* 2013; Fukuyama *et al.* 2013) have placed SUMO-mediated regulation as an important PTM for TL signaling. For IMD/NF-κB signaling Fukuyama *et al.* (2013) identified 369 proteins as part of the “IMD interactome”; 120 proteins from our unique list are present in their dataset. Our study implicates Casp as a definitive SUMO target whereas IMD and REL activity also may be conjugated by SUMO. Previously, IRD/IKKβ SUMOylation has been shown to be critical for IMD signaling (Fukuyama *et al.* 2013). Also enriched in our list are SkpA, a member of the SCF ubiquitin ligase complex that regulates REL stability as well as Ubiqutin E2 conjugases Uev1a and Effete, both important for Dredd activation via K63 polyubiquitination (Figure 6D). The JNK pathway is an important regulator of the immune response, and we find that Jra and Kay are validated SUMO targets with Basket a potential target. Jra and Kay heterodimerize to form the important transcriptional regulator AP1 that regulates a number of downstream genes, including NF-κBs. STAT92E, the effector of the JAK-STAT pathway, has been shown previously to be SUMOylated. P38b, in the MAPK pathway is a validated target and Pirk, which is activated by p38b/MAPK signaling is a negative regulator of IMD signaling. In addition to the signaling pathways that are involved in immune signaling, our list suggests important roles for MARS complex in immune signaling and also proteins involved in trafficking (Table S1 and Table S2), as are adaptor proteins, such as 14-3-3ε and 14-3-3ζ.

An obvious experiment would be to measure the differential ability of Casp(wt) and Casp(K551R) to modulate IMD signaling in S2 cells. Casp(wt), however, appears not to regulate AMPs expression when expressed in S2 cells, even when different tags (6X-His, HA) are tested at both N and C terminal to the molecule. Also, studies in which REL cleavage can be monitored postexpression of Casp and Casp(K551R) are inconclusive because of large variability of cleavage kinetics in biological replicates. Because Casp originally was identified as a negative regulator in animal studies (Kim *et al.* 2006), we plan to test biologic activity of Caspar variants in flies and are in the process of generating transgenic flies for future experiments.

In summary, our methodology, which involves measurement of changes to the SUMO enriched-proteome using MS, followed by physical demonstration of SUMOylation by *in bacto* system, is a robust method for detecting SUMO pathway proteins that respond to immune challenge. SUMOylation appears to be widespread in the *Drosophila* proteome, with specific roles in immune response. SUMOylated proteins can be explored for conjugation sites by lysine mutagenesis and mutants tested *in vivo*. A complete understanding of roles for SUMO modification in the immune response will emerge when we have quantitative data for immune modulation by each SUMOylated substrate. The global effect of SUMO modulation would be a complex integral of each individual SUMOylation/ deSUMOylation event. We have contributed to understanding of immune regulation by describing the LPS-responsive, SUMO-enriched proteome in *Drosophila*. The proteins in our list represent a small fraction (<5%) of the protein coding genes in *Drosophila*. The hits do not overlap significantly with the immune transcriptome, confirming independent roles for dynamic, post-translational modifications in immune regulation.

## 
